# Supplementary material for: Combined Computer-Aided Predictors to Improve the Thermostability of Nattokinase
Source: Foods. 2023 Aug 14;12(16):3045. doi: 10.3390/foods12163045 (PMC10453301; doi:10.3390/foods12163045)
Supplement: Supplementary file 1 [file foods-12-03045-s001.zip › foods-2537819-supplementary.pdf]

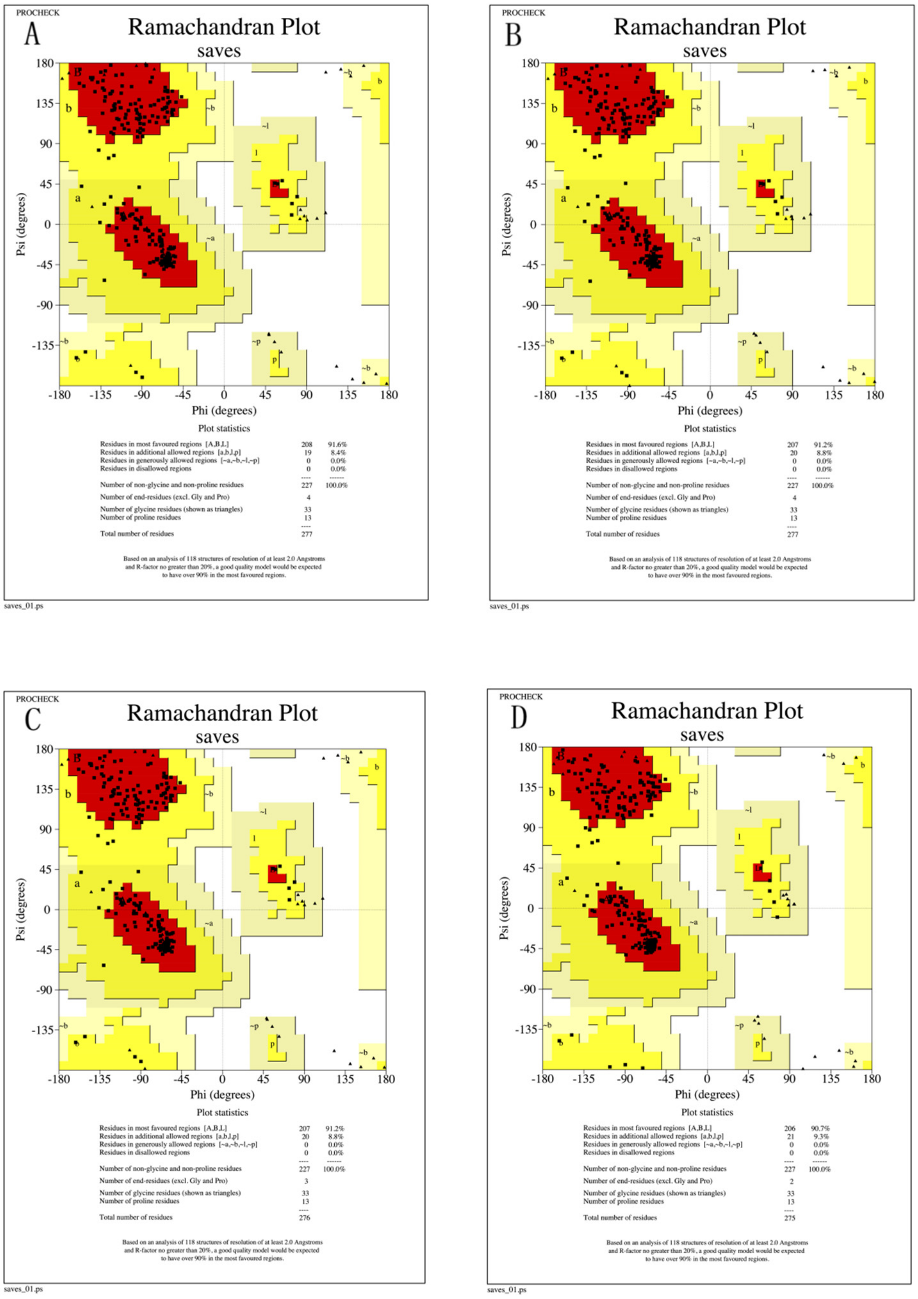

Figure S1 . Ramachandran plots of rAprY and its variants: (A) wild type rAprY; (B) S33T; (C) T174V; (D)S33T-T174V. Residues in the most favored regions are shown in red. Residues in additionally allowed regions are shown in yellow.

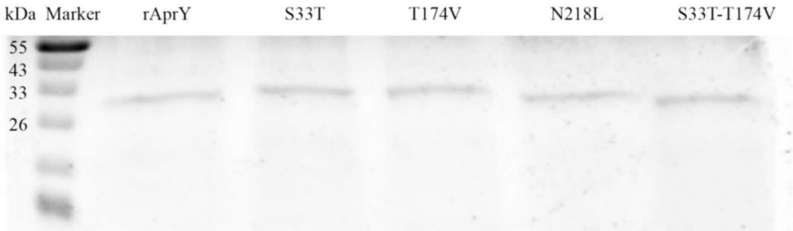

Figure S2. SDS-PAGE of purified rAprY and its variants. Lane Marker: Standard marker protein with molecular weights 14.3–200 kDa. The arrow indicates the corresponding bands of mature enzymes.

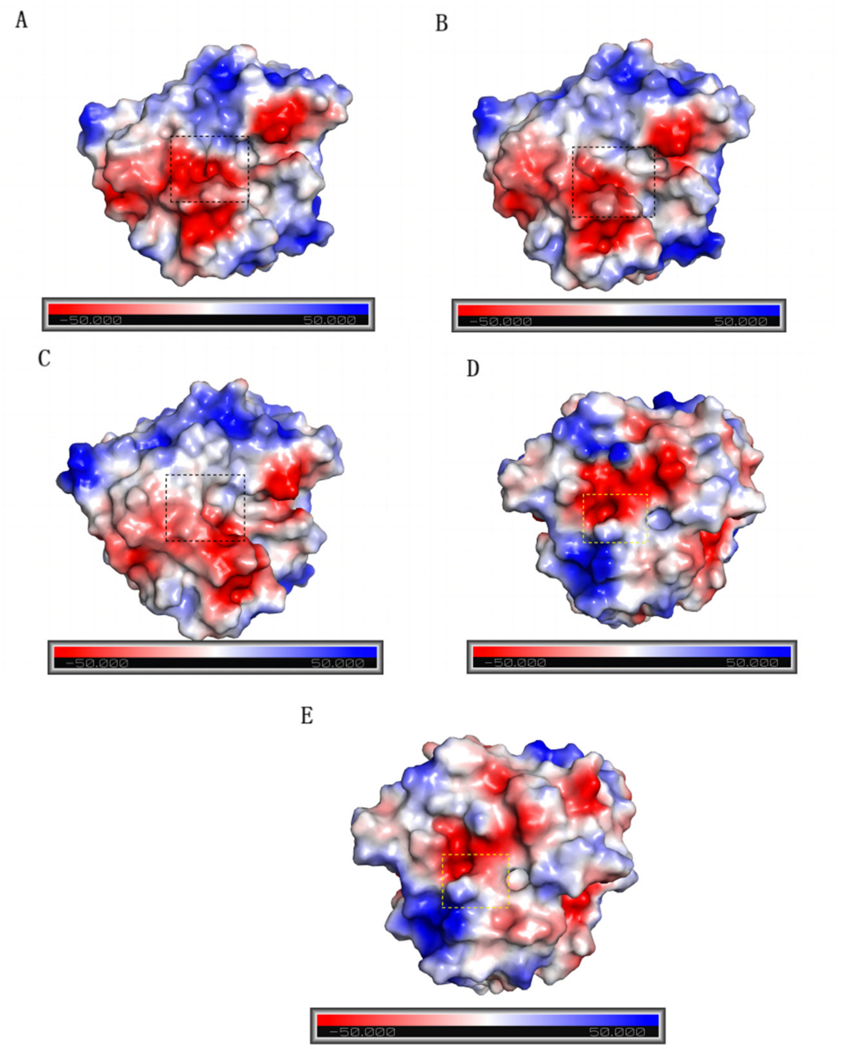

Figure S3. Surface charge analysis of rAprY and its variants. (A) and (D): rAprY; (B) S33T; (C) N218L; (E) T174V; The catalytic center region of the enzyme is marked by a black dashed box; The region of amino acid residue 174 is marked by yellow dashed boxes.

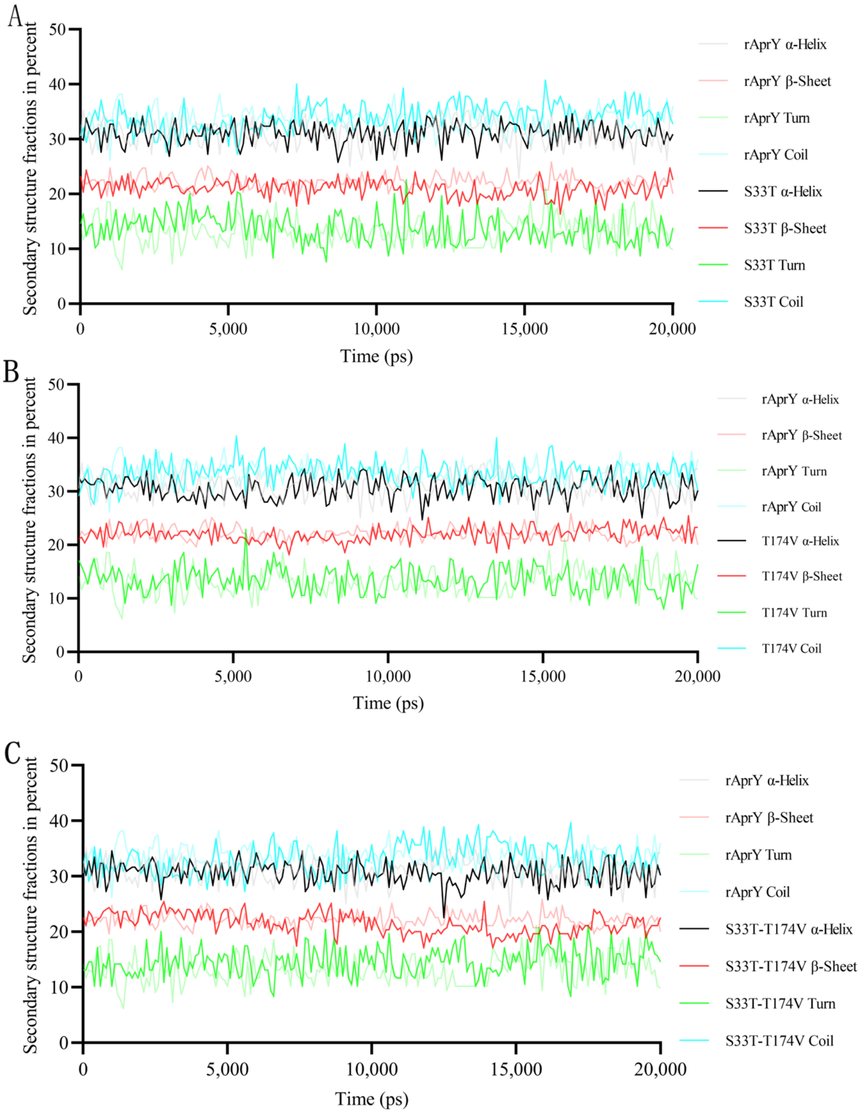

Figure S4. Protein secondary structure content (vertical axis) as a function of simulation time (horizontal axis). (A) rAprY and S33T; (B) rAprY and T174V; (C) rAprY and S33T-T174V.

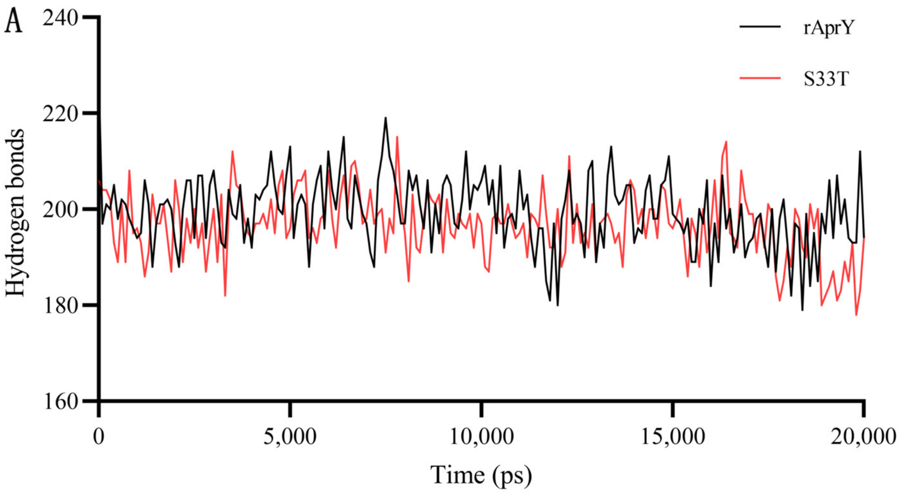

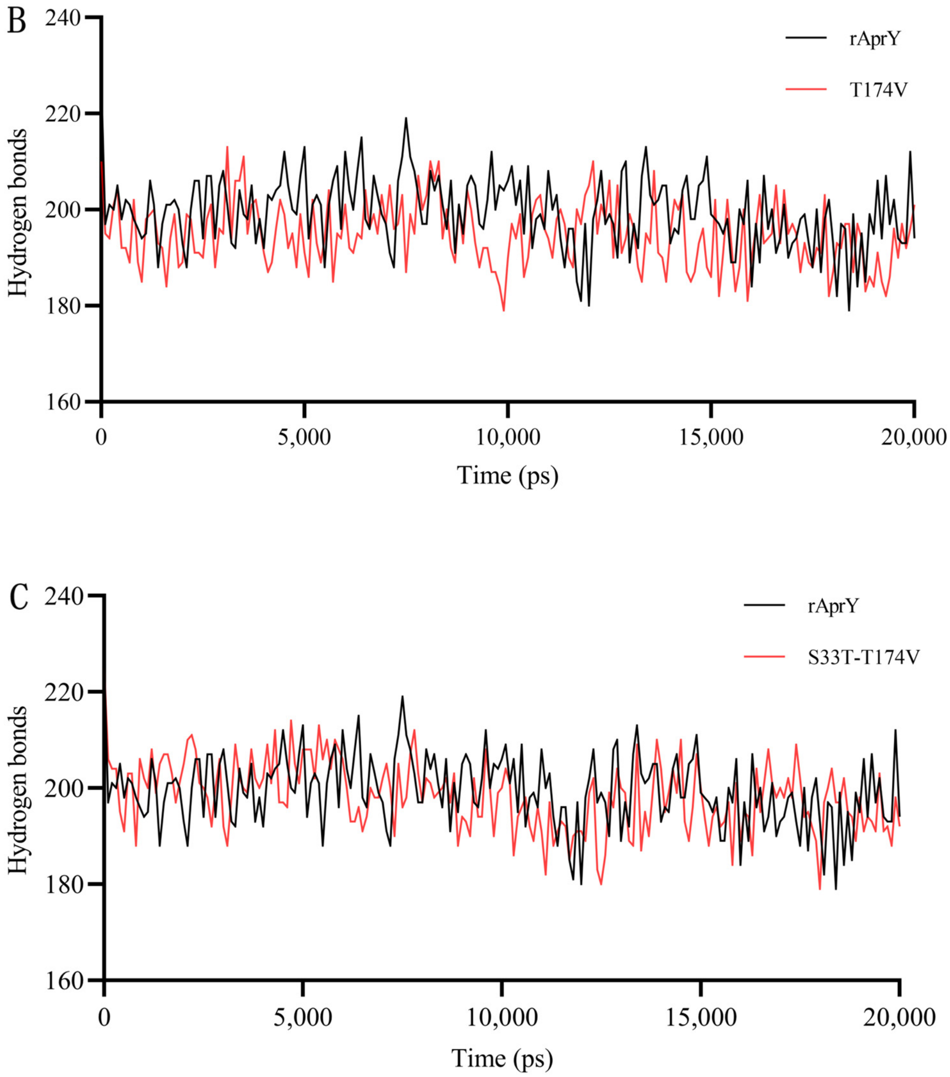

Figure S5. Number of hydrogen bonds (vertical axis) as a function of simulation time (horizontal axis). (A) rAprY and S33T; (B) rAprY and T174V; (C) rAprY and S33T-T174V.

Table S1 Primers used for site-directed mutagenesis.

Primer name	Gene sequence（5′-3′）
S204M-up	atggctcctggcgtgatgatccaaagca
S204M-down	catcacgccaggagccattacatcaagc
S105L-up	ggaagcggccaatatctatggattatta
S105L-down	tagatattggccgcttcctgttgaatca
S89I-up	acaaacgcgcaagtcattgatcgtttag
S89I-down	tattgcgcttggcgctacgcccagaaca
R247I-up	acaaacgcgcaagtcattgatcgtttag
R247I-down	aatgacttgcgcgtttgtccaagtcggg
S204L-up	atggctcctggcgtgttaatccaaagca
S204L-down	taacacgccaggagccattacatcaagc

H226F-up	tccatggcgactcctttcgttgccggag
H226F-down	gaaaggagtcgccatggacgttccgtta
S53I-up	gcaagcttcgttcctattgaaacaaacc
S53I-down	aataggaacgaagcttgctccgcctctg
N218M-up	acttacggcgcttatatgggaacgtcca
N218M-down	catataagcgccgtaagtgcctccagga
N218L-up	acttacggcgcttatctaggaacgtcc
N218L-down	tagataagcgccgtaagtgcctccagga
N25G-up	ggctacacaggctctggcgtaaaagtag
N25G-down	gccagagcctgtgtagccttgagagtga
S33T-up	gtagctgttatcgacaccggaattgact
S33T-down	ggtgtcgataacagctacttttacgtta
S63G-up	taccaggacggcagtggtcacggtacgc
S63G-down	accactgccgtcctggtatgggtttgtt
V121I-up	tccaacaatatggatattatcaacatga
V121I-down	aatatccatattgttggaaatggcccac
A150V-up	agcggtatcgtcgttgttgccgcagccg
A150V-down	aacaacgacgataccgctggaaaccgct
T174V-up	gcaaaatatccttctgttattgcagtag
T174V-down	aacagaaggatattttgcagggtagaag
N181D-up	gcagtaggtgcggtagacagcagcaacc
N181D-down	gtctaccgcacctactgcaatagtagaa
G110P-up	agctggattattaaccccattgagtggg
G110P-down	ggggttaataatccagctatattggccg
A114P-up	aacggcattgagtggcccatttccaaca
A114P-down	gggccactcaatgccgttaataatccag
